# Oxytocin Protects Against Corticosterone-Induced DA Dysfunction: An Involvement of the PKA/CREB Pathway

**DOI:** 10.1007/s11064-024-04294-7

**Published:** 2024-11-28

**Authors:** Sirinun Chaipunko, Tichaporn Sookkua, Chutikorn Nopparat, Nuanchan Chutabhakdikul

**Affiliations:** 1 Research Center for Neuroscience, Institute of Molecular Biosciences, Mahidol University, Nakhon Pathom, 73170 Thailand; 2 Faculty of Physical Therapy, Mahidol University, Nakhon Pathom, 73170 Thailand; 3 Faculty of Physical Therapy, Saint Louis College, Bangkok, 10120 Thailand; 4 Innovative Learning Center, Srinakharinwirot University, Bangkok, 10110 Thailand

**Keywords:** Oxytocin, Stress, Dopamine, Tyrosine hydroxylase, PKA/CREB, SH-SY5Y cells

## Abstract

Chronic stress disrupts dopamine (DA) transmission, adversely affecting mood and contribution to neuropsychiatric disorders like ADHD, autism, schizophrenia, anxiety, depression, and drug addiction. The neuropeptide oxytocin (OXT) plays a key role in social cognition, bonding, attachment, and parenting behaviors. In addition, OXT can modulate the activity of the HPA axis, counteracting the effects of stress, and alleviating fear and anxiety. However, whether OXT can mitigate stress-induced DA dysfunction and the underlying mechanisms remains unclear. This study investigated the neuroprotective effects of OXT on corticosterone (CORT) induced DA dysfunction in the neuroblastoma cell line SH-SY5Y. The results revealed that CORT decreases the levels of intracellular signaling molecules associated with DA function, including phosphorylated tyrosine hydroxylase (pTH), phosphorylated cAMP response element-binding protein (pCREB), and protein kinase A (PKA). Interestingly, pretreatment with OXT mitigated CORT-induced DA dysfunction through its potent PKA activator properties. In addition, the neuroprotective effect of OXT was abolished by atosiban (an OXT receptor antagonist) or H89 (a PKA inhibitor). Our results suggest that OXT protects dopaminergic neuroblastoma cells from CORT-induced DA dysfunction, potentially through the involvement of oxytocin receptors and the PKA/CREB signaling pathway. These findings contribute to the understanding of the neurobiological mechanisms underlying stress resilience and highlight potential pathways for developing targeted treatments that leverage the neuroprotective properties of OXT to address disorders characterized by DA dysregulation and impaired stress responses.

## Introduction

Stress activates the hypothalamus-pituitary-adrenal (HPA) axis, resulting in corticosteroid (CORT) secretion. Normally, this helps restore homeostasis [1]. However, chronic stress dysregulates the HPA axis, leading to persistently elevated CORT levels and contributing to mental illness and stress-related brain disorders [1–3]. Stress has a significant impact on dopamine function, which is crucial for mental health. Chronic stress can lead to alterations in dopamine signaling pathways, potentially contributing to the development of mental disorders such as depression, anxiety, and schizophrenia [4]. Understanding these changes is essential for developing effective interventions and treatments for stress-related mental health issues.

Dopamine (DA) has diverse functions across various brain regions. In the prefrontal cortex, DA regulates cognition and executive functions such as working memory, attention, and decision-making [5–8]. In the limbic system, DA is crucial for processing rewards, pleasure, motivation, and reinforcement learning [8, 9]. Impairments in DA function are associated with learning and memory deficits and contribute to psychiatric conditions such as schizophrenia, autism, ADHD, and drug addiction [8, 10]. Evidence for DA dysfunction includes disturbances in tyrosine hydroxylase (TH) activity. Animal models have shown a decrease in TH expression in the substantia nigra and the striatum in ADHD-induced rats [11]. Additionally, selectively lesioning the striatum and affecting TH-expressing interneurons leads to impaired goal-directed behavior in mouse models [12].

Stress negatively impacts mesolimbic dopamine function and affects social behaviors [13]. Chronic stress, for example, is strongly associated with disrupted dopamine signaling in the medial prefrontal cortex and ventral striatum, affecting reward sensitivity [14] and reward learning [15]. Recent findings by Quessy et al. (2021) revealed that chronic stress induces morphological and molecular changes in dopaminergic neurons in both the mesolimbic and mesocortical pathways. These changes are accompanied by a significant reduction in dopamine synthesis in the ventral striatum, contributing to depressive-like behaviors in rodent models [16, 17]. These lines of evidence suggest that stress disrupts dopamine transmission, leading to adverse effects on mood and behavior. Additionally, stress is a risk factor that can trigger the initiation, escalation, and relapse of drug addiction through dopaminergic mechanisms [16].

Oxytocin (OXT) is a neuropeptide hormone crucial for various physiological processes. Peripherally, OXT facilitates newborn delivery, milk ejection, and parenting behaviors [18–20]. Centrally, OXT is associated with higher-order cognitive processes such as social attachment and bonding [21]. Additionally, OXT administration has shown potential for improving social deficits in animal models of autism and schizophrenia [21, 22]. Consequently, OXT is considered a promising neuromodulator for therapeutic strategies targeting stress-induced psychiatric disorders and mental illnesses, which may result from chronic stress influencing dopaminergic dysregulation [23]. Interestingly, OXT administration reportedly enhances attention and social behavior and reduces stress [24–26]. Although OXT is a critical modulator of HPA axis activity and related neural pathways [26], the mechanisms underlying its modulation of stress-induced dopamine neuron dysfunction remain unclear. Various stressful stimuli activate oxytocin neurons and facilitate OXT release, modulating the stress response in animal model studies. This process involves OXT receptor activation in several distinct brain regions [27, 28].

Oxytocin and dopamine share similarities in their organization and projection sites. For example, DA receptors are present in OXT neurons, and OXT receptors are found in the ventral tegmental area (VTA) and substantia nigra pars compacta (SNc). OXT appears to directly increase the activity of VTA neurons and indirectly inhibit SNc dopaminergic neurons. OXT and DA synergistically facilitate social behavior through the direct projections of VTA dopaminergic neurons to the ventral hippocampus and nucleus accumbens [29]. These findings indicate that OXT modulates the activity of dopamine neurons in the VTA and substantia nigra, playing a crucial role in social behavior modulation [30]. Understanding the mechanisms by which dopamine signaling interacts with the oxytocinergic system can be valuable for developing therapeutic strategies for conditions involving social deficits.

The potential neuroprotective effect of OXT in mitigating the negative impact of stress on dopaminergic neurons and its underlying mechanisms are not yet fully understood and require further exploration. However, additional research is needed to investigate the signaling mechanisms through which OXT counteracts the toxic effects of CORT on dopaminergic function in vitro. Human neuroblastoma SH-SY5Y cells exhibit neuroblast-like characteristics similar to those of catecholaminergic neurons and express markers such as tyrosine hydroxylase (TH) and dopamine-β-hydroxylase (DBH) [31–33]. To elucidate the intricate processes and signaling mechanisms through which OXT influences dopaminergic activity following exposure to a toxic dose of CORT, this study utilized the SH-SY5Y neuroblastoma cell line to investigate the effects of CORT on TH and CREB phosphorylation. We hypothesize that OXT can protect against corticosterone-induced neurotoxicity in SH-SY5Y cells possibly through the oxytocin receptor and the PKA/CREB signaling pathway.

## Materials and methods

### Cell Culture

Human neuroblastoma SH-SY5Y cells were cultured at a density of 1.5 × 10^5^ cells/ml in basal medium supplemented with Dulbecco’s modified Eagle’s medium (DMEM) supplemented with L-glutamine, sodium pyruvate, phenol red, 100 U/ml penicillin, 100 µg/ml streptomycin, and 10% Gibco^®^ fetal bovine serum (all from Invitrogen, USA). The cells were incubated at 37 °C in a humidified atmosphere with 5% CO2. Once the cells reached confluency, they were passaged using gentle trypsinization with 0.25% trypsin-EDTA containing phenol red to detach the adhered cells. Trypsinization was halted by adding an equal volume of DMEM supplemented with 10% FBS. The culture medium was replaced with fresh medium every 24 h.

### Drugs and Reagents

Corticosterone (CORT; product no. C2505), oxytocin (OXT; product no. O3251), a PKA inhibitor (H89; product no. B1427), and an OXT receptor antagonist (atosiban; product no. A3480) were obtained from Sigma‒Aldrich (St. Louis, MO, USA). The antibodies used for verification in this study included a βIII-tubulin antibody (product no. SAB4500088), which was purchased from Sigma‒Aldrich; an anti-β-actin antibody (cat. no. SC-69879); and a monoclonal mouse anti-total tyrosine hydroxylase antibody (tTH, cat. no. SC-25269), which was purchased from Santa Cruz Biotechnology (Santa Cruz, CA, USA). Polyclonal rabbit anti-phospho-tyrosine hydroxylase (Ser40) antibody (pTH, Cat. No. 2791 S), monoclonal rabbit anti-phospho-cAMP-response-element-binding-protein (Ser133) antibody (pCREB; Cat. No. 9198 S), and polyclonal rabbit anti-catalytic-protein kinase A-α subunit antibody (PKAc-α; (Cat. No. 4782 S) were purchased from Cell Signaling Technology (Beverly, MA, USA). CORT was dissolved in dimethyl sulfoxide (DMSO), which was also acquired from Sigma‒Aldrich. All cell culture reagents were purchased from Invitrogen (Carlsbad, CA, USA).

### Cell Treatment

**Fig. 1:**
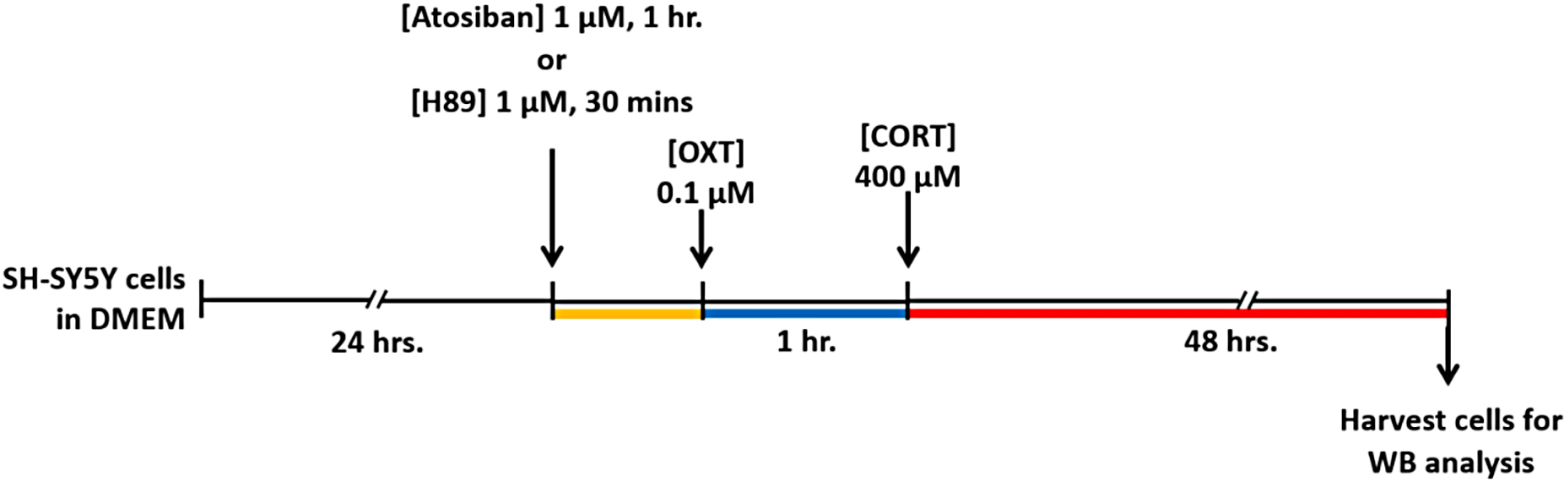
Experimental design

SH-SY5Y cells were seeded into 6 mm Petri dishes in DMEM supplemented with 10% FBS for 24 h to study CORT-induced neurotoxicity and its effects on TH and CREB phosphorylation as well as PKA activity. The cells were treated with 400 µM CORT for 48 h. To assess whether OXT could protect cells from CORT-induced toxicity, cells were pretreated with 1 µM atosiban (an OTR antagonist) for 1 h, followed by 0.1 µM OXT for another hour before exposure to CORT. To investigate the involvement of PKA in OXT-mediated protection, cells were pretreated with 1 µM H89 for 30 min, followed by 0.1 µM OXT and subsequent CORT treatment (see Fig. 1 for the experimental design). All the experiments were conducted with a sample size of 4 (*n* = 4), and each experiment was performed in duplicate.

### Western Blot/Immunoblotting

After the cells were treated under various experimental conditions, they were collected and washed with cold 0.1 M PBS. The tissues were then homogenized and sonicated in lysis buffer containing 0.1 M NaCl, 0.01 M Tris-HCl (pH 7.6), 0.001 M EDTA (pH 8.0), 1 µg/ml aprotinin, 100 µg/ml phenylmethylsulfonyl fluoride (PMSF), and protease and phosphatase inhibitors. Following sonication, the samples were centrifuged at 14,000 rpm for 20 min. The supernatant, containing the soluble protein, was subjected to Western blot analysis. Protein concentrations were determined using a BCA Protein Assay Kit (Pierce, Rockford, IL) and a 96-well microplate reader (Spectra Max 340; Molecular Devices, Sunnyvale, CA). Samples containing 25 µg of protein were electrophoresed on a 10% SDS‒polyacrylamide gel and transferred to polyvinylidene fluoride (PVDF) membranes. The membranes were blocked with either 5% nonfat milk or 3% bovine serum albumin (BSA) in TBST at room temperature for 1 h. Subsequently, the PVDF membranes were incubated with the appropriate primary antibodies at 4 °C overnight. After washing three times with TBST, the membranes were incubated with horseradish peroxidase (HRP)-conjugated secondary antibodies for 1 h at room temperature. The membranes were then washed to remove any nonspecific binding of secondary antibodies. Finally, the labeled protein bands were detected using Amersham ECL Prime Western Blotting Detection Reagent (G&E Healthcare Bio-Sciences, Pittsburgh, USA) and visualized with a Bio-Rad^®^ chemiluminescence detection system (Bio-Rad^®^ ChemiDoc™ XRS^+^ System). The values are presented as the percentage change in the optical density of each experimental condition relative to the sham control within individual blots.

In this study, changes in the TH enzyme are presented as a ratio of phosphorylated TH (pTH) to total TH (tTH), as total TH levels can vary significantly across experimental conditions or cell types. This pTH/tTH ratio distinguishes phosphorylation effects from changes in protein expression. In contrast, the functionality of the CREB transcription factor is primarily attributed to its phosphorylated form (pCREB), particularly phosphorylation at serine 133, which is essential for its transcriptional activation [34].

According to Rosethorne et al. (2008), total CREB levels in undifferentiated SH-SY5Y neuroblastoma cells generally remain at basal levels, supporting basic cellular functions like survival and signaling, with no significant changes observed across various treatment conditions [35]. Similarly, differentiated SH-SY5Y cells also showed no significant differences in total CREB under different treatment conditions [36, 37]. Therefore, in this study, we emphasize pCREB to capture its functional state, as pCREB levels alone are more relevant to understanding CREB’s role in our experimental context.

### Statistical Analysis

The data were calculated from 4 independent experiments, each conducted in duplicate. The data are presented as the means ± SEMs. Statistical comparisons were conducted using one-way analysis of variance (ANOVA) followed by the Newman‒Keuls multiple comparison test. A probability value of *p <* 0.05 was considered to indicate statistical significance. All the statistical analyses were performed using GraphPad Prism^®^ software version 8.0.1.

## Results

### Determine the Optimal Doses of Drugs Treatment

**Fig. 2:**
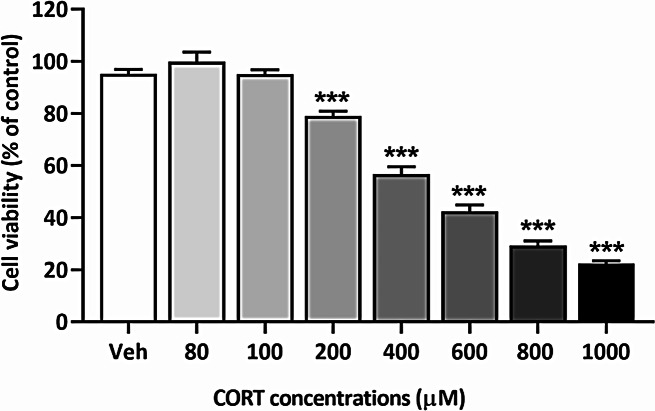
Effect of CORT on SH-SY5Y cell viability. SH-SY5Y cells were exposed to increasing doses of CORT for 48 h. Cell viability was assessed using the MTT assay, showing a dose-dependent decrease in survival rate. Data are presented as mean ± SEM from 3 independent experiments, each performed in triplicate. Statistical significance is indicated as ***p < 0.001 compared to the vehicle group

To examine the cytotoxic effect of corticosterone (CORT) on SH-SY5Y cells, the cells were exposed to varying concentrations of CORT (0, 80, 100, 200, 400, 600, 800, and 1,000 µM) for 48 h. Cell viability was assessed using the MTT assay (see Fig. 2). The results demonstrated a dose-dependent reduction in cell viability, with higher concentrations of CORT leading to progressively greater cytotoxic effects. Based on these findings, 400 µM was chosen as the optimal dose for further experiments, as it induced a significant yet submaximal reduction in viability, making it suitable for investigating potential protective effects.

**Fig. 3:**
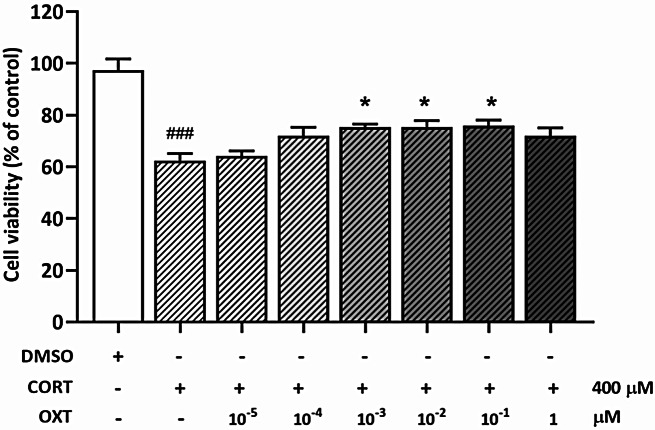
Cell viability assay for the protective effect of OXT against CORT-induced toxicity in SH-SY5Y cells. SH-SY5Y cells were pretreated with increasing doses of OXT for 1 h before being exposed to 400 µM CORT for 48 h. Cell viability was assessed using the MTT assay. The data are presented as mean ± SEM from 3 independent experiments, each performed in triplicate. Statistical significance is indicated as *p < 0.05 compared to CORT alone, and ^###^*p <* 0.001 compared to the vehicle group

To determine whether OXT could protect against CORT-induced cytotoxicity, SH-SY5Y cells were pretreated with increasing doses of OXT (0, 10⁻⁵, 10⁻⁴, 10⁻³, 10⁻², 10⁻¹, and 1 µM) for 1 h before exposure to 400 µM CORT for 48 h (see Fig. 3). The MTT assay revealed a dose-dependent protective effect of OXT, with higher doses of OXT significantly improving cell viability compared to CORT treatment alone. The optimal dose of OXT for protection was found to be 10⁻¹ µM, as it provided the most significant improvement in cell viability. These findings suggest that OXT may counteract the toxic effects of CORT, potentially through mechanisms that support cell survival under stress conditions. Statistical analysis confirmed the significance of these protective effects, with *p <* 0.05, indicating the efficacy of OXT in enhancing cell viability. Additionally, a dose of 1.0 µM was chosen for both atosiban and H89 based on previous studies that identified this concentration as optimal for similar experimental conditions [37–40].

### Oxytocin Protects against CORT-Induced DA Dysfunction in SH-SY5Y Cells

**Fig. 4:**
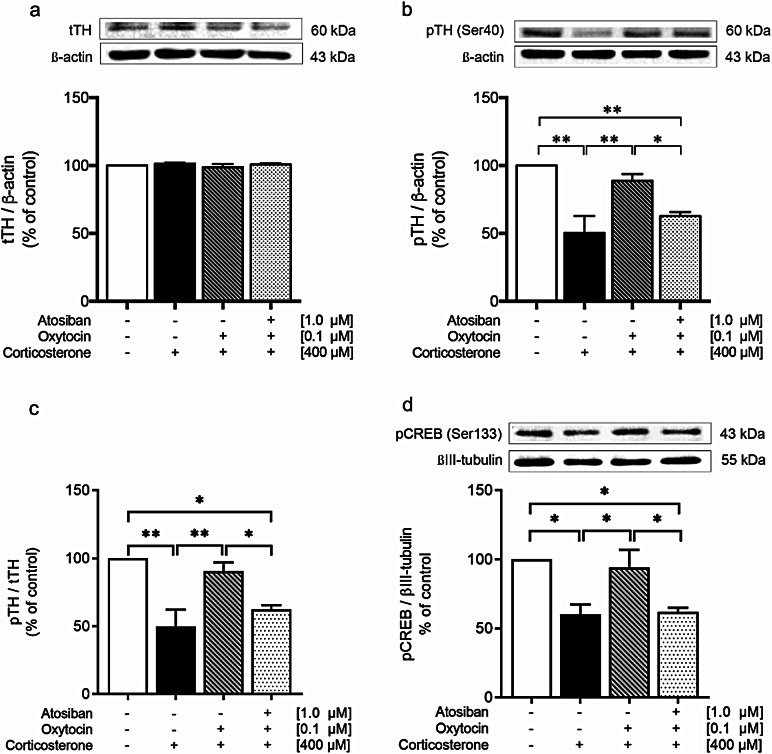
Western blot analysis of tTH (**a**), pTH (**b**), pTH/tTH (**c**), and pCREB (**d**) expression in control, CORT-treated, OXT-pretreated, and atosiban + OXT-pretreated SH-SY5Y cells (*n* = 4 per group). The intensity of the product bands was normalized against that of the respective internal controls. Bar graph displays the corresponding quantitative results. The data are expressed as a percentage of the control. The values represent means ± SEMs. Statistical significance is denoted as *p < 0.05 and **p < 0.01

This study explored the protective effects of OXT against CORT-induced DA dysfunction in SH-SY5Y cells. Forty-eight hours of exposure to a toxic dose of CORT (400 µM) led to a significant reduction in the pTH/tTH ratio by 49.67% compared to the control group (*p <* 0.01; Fig. 4a). However, pretreatment with OXT (0.1 µM) for 1 h reversed this effect, as shown by increasing the pTH/tTH ratio to 90.38% of the control group, and significant increase compared to the CORT-treated group (*p <* 0.01). This protective effect of OXT was attenuated by pretreatment with OXT receptor antagonist, atosiban. Pretreatment with 1.0 µM atosiban significantly decreased the pTH/tTH ratio as compared to the control group (*p <* 0.05) and OXT pretreatment group (*p <* 0.05). Moreover, there was no significant difference in the pTH/tTH ratio between the atosiban-treated group and the CORT-treated group.

In addition, OXT can protect against CORT-induced alterations in pCREB. Treatment of SH-SY5Y cells with a toxic dose of CORT for 48 h led to a significant decrease in pCREB levels by 60.18% compared to those in the control group (*p <* 0.05; Fig. 4b). Nonetheless, pretreatment with 0.1 µM OXT for 1 h effectively reversed this reduction, leading to a significant increase in pCREB levels compared to those in the CORT-treated group (*p <* 0.05). The protective effect of OXT was compromised by pretreatment with the OXT receptor antagonist atosiban. Pretreatment with 1.0 µM atosiban notably reduced pCREB levels to those observed in the CORT-treated group. The atosiban-treated group exhibited a significant decrease in pCREB levels compared to those in the control (*p <* 0.05) and OXT-pretreated groups (*p <* 0.05). These findings indicate that CORT reduces TH and CREB phosphorylation but that this effect can be prevented by pretreatment with OXT. Additionally, the protective effects of OXT against CORT-induced decreases in pTH and pCREB occur through the OXTR signaling pathway.

### OXT Counteracts CORT-Induced DA Dysfunction through a PKA-Dependent Mechanism

**Fig. 5:**
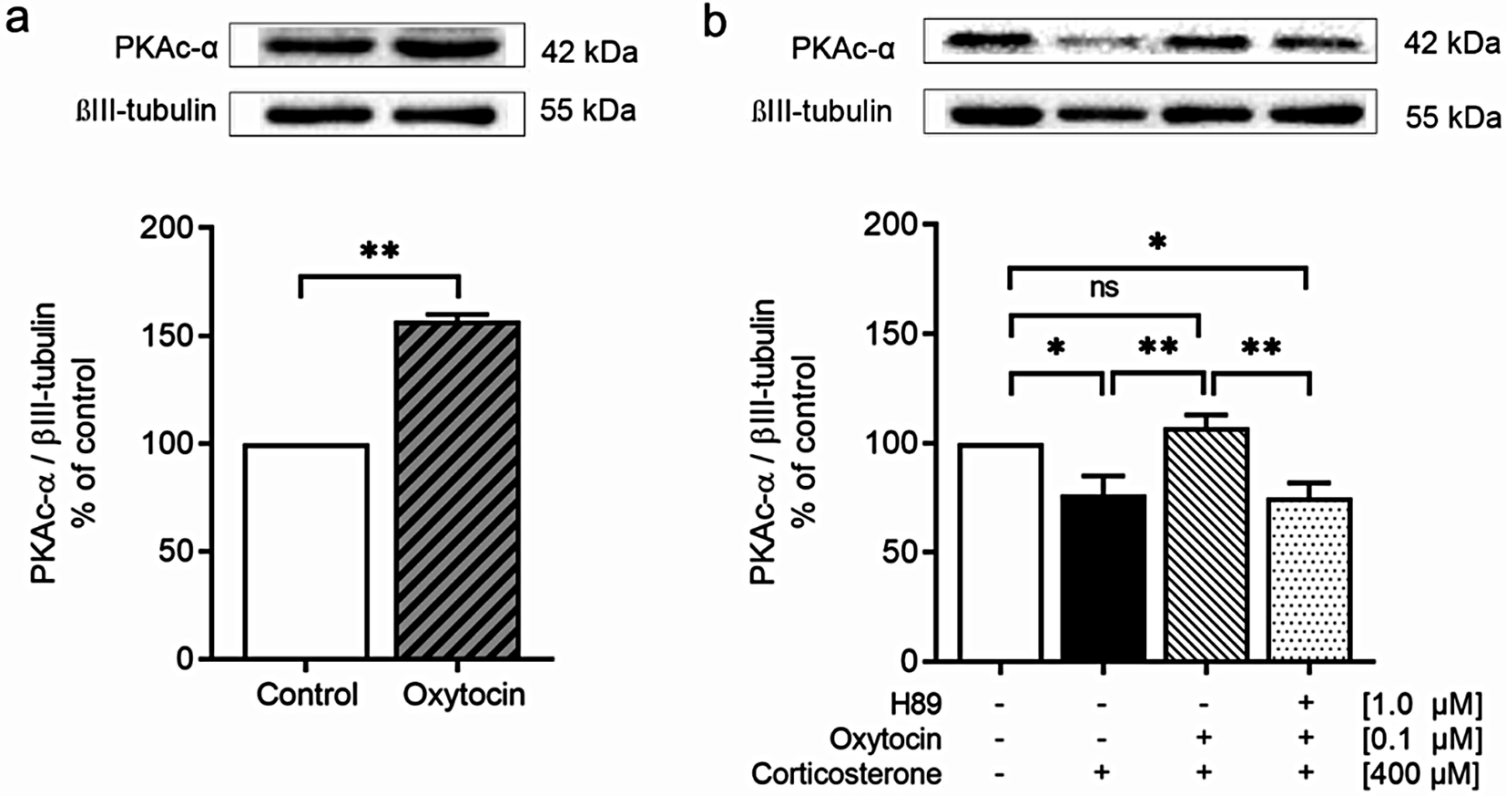
(**a**) Western blot analysis of PKAc-α in control and OXT-treated SH-SY5Y cells (*n* = 4 per group) and (**b**) Western blot analysis of PKAc-α in control, CORT-treated, OXT-pretreated, and H89 + OXT-pretreated SH-SY5Y cells (*n* = 4 per group). The intensity of the product bands was normalized against that of the βIII-tubulin band as an internal control. The bar graph displays the corresponding quantitative results. The data are expressed as a percentage of the control. The values represent the means ± SEMs. Statistical significance is denoted as *p < 0.05, **p < 0.01, and ns = not significant difference

To test the hypothesis that OXT pretreatment can prevent CORT-induced DA dysfunction via the PKA signaling pathway, we first examined whether OXT affects the level of PKAc-α, a key signaling molecule that can phosphorylate the CREB protein. The results showed that OXT is a powerful PKA activator. OXT alone significantly increased PKAc-α levels to 150% of the control level (*p <* 0.01; Fig. 5a). Therefore, we further investigated the protective effects of OXT against CORT-induced alterations in PKAc-α in SH-SY5Y cells. Exposure to a toxic dose of CORT (400 µM) for 48 h significantly decreased PKAc-α levels compared to those in the control group (*p <* 0.05). However, OXT pretreatment (0.1 µM) for 1 h effectively reversed this reduction by inducing a significant increase in PKAc-α levels compared to that in the CORT-treated group (*p <* 0.01); moreover, there was no significant difference compared with that in the control group. Furthermore, pretreatment with 1.0 µM H89 significantly reduced PKAc-α to the level observed in the CORT-treated group. The H89-treated group exhibited significantly lower PKAc-α levels than did the control (*p <* 0.05) and OXT-pretreated groups (*p <* 0.01) (see Fig. 5b). Our finding that the protective effects of OXT against the CORT-induced decrease in PKAc-α were abolished by the PKA inhibitor H89 provides important mechanistic insights into the molecular interaction between CORT and OXT. This finding suggested that OXT plays a direct regulatory role on PKAc-α in the presence of CORT.

**Fig. 6:**
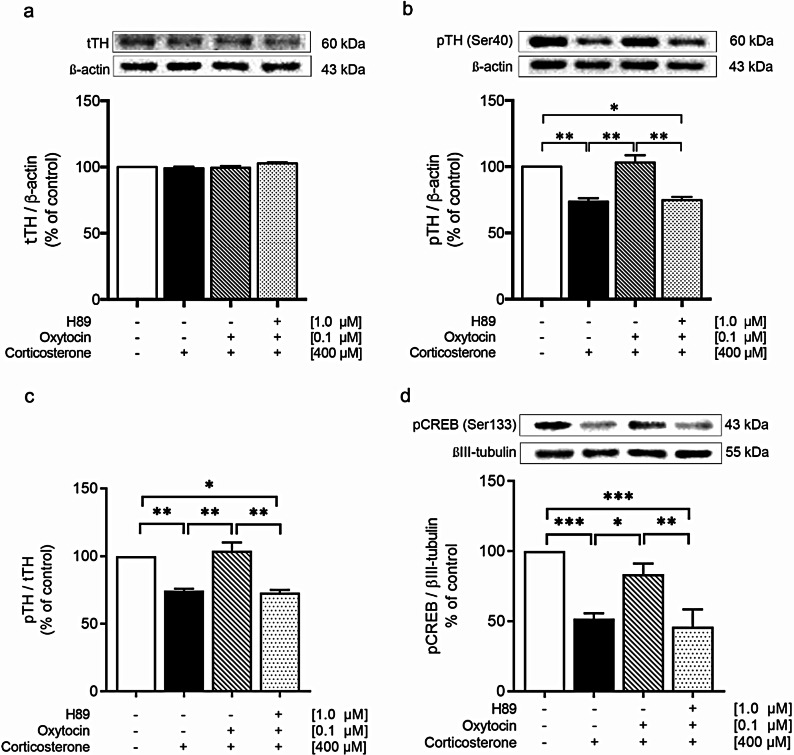
Western blot analysis of tTH (**a**), pTH (**b**), pTH/tTH (**c**) and pCREB (**d**) in control, CORT-treated, OXT-pretreated, and H89 + OXT-pretreated SH-SY5Y cells (*n* = 4 per group). The intensity of the product bands was normalized against that of the respective internal controls. Bar graph displays the corresponding quantitative results. The data are expressed as a percentage of the control. The values represent the means ± SEM. Statistical significance is denoted as *p < 0.05, **p < 0.01, *** *p <* 0.001

Finally, we tested the hypothesis that OXT counteracts the CORT-induced decrease in pTH and pCREB levels through a PKA-dependent mechanism by pretreating SH-SY5Y cells with the PKA inhibitor H89. The results revealed that H89 significantly reversed the protective effect of OXT by reducing the pTH (*p <* 0.01; Fig. 6a) and pCREB (*p <* 0.01; Fig. 6b) levels to those of the CORT-treated group, similar to what was observed under CORT treatment alone. These findings indicate that the phosphorylation of TH and CREB is mediated by PKA through OXTR signaling transduction in response to stress-induced DA dysfunction.

## Discussion

Dopamine dysfunction plays a significant role in various disorders characterized by social impairment, including ADHD, ASD, schizophrenia, anxiety, and depressive disorders [9, 15, 41, 42]. Furthermore, stress can negatively impact DA function, affecting mood and social cognition. A common thread across these disorders is the impact of altered DA signaling on social cognition, motivation, and behavior under stress conditions [13, 16, 17, 43]. Previous studies utilizing various stress induction models have also shown alterations in TH activity following stress. These stress models, which include social defeat, immobilization, foot shock, and hypoglycemia induction, decrease TH phosphorylation in the brain [44–47]. The phosphorylation of the TH enzyme serves as a key indicator of dopaminergic function. Decreased TH phosphorylation can lead to DA synthesis failure and DA hypofunction [48]. In addition, the TH gene has been associated with the pathogenesis of ASD and ADHD, particularly concerning executive function performance under stressful conditions [41, 42].

Transcription of the TH gene is CREB dependent [49]. CREB is a critical transcription factor implicated in neural plasticity, underpinning memory and learning. The phosphorylation of CREB at Ser133 is essential for the induction of TH gene transcription via CREB/AP-1 family members in PC12 cells [50]. In this study, the finding that CORT decreases pCREB aligns with the findings of previous studies showing that chronic stress reduces CREB phosphorylation, leading to decreased neural plasticity in limbic brain areas associated with mood disorders [51]. Additionally, severe stress adversely affects CREB and BDNF in the developing hippocampus and other brain regions, further impacting neural plasticity [52]. Our results indicate that CORT might disrupt DA synthesis and induce DA dysfunction by reducing pCREB and pTH levels in SH-SY5Y cells. These findings highlight that DA dysfunction might be a key mechanism underlying the effects of stress on mood and social dysfunction.

Research indicates that OXT reduces stress responses in brain regions involved in regulating HPA axis activity, such as the paraventricular nucleus (PVN), ventrolateral septum (LSV), and dorsal hippocampus [53]. Moreover, OXT has been shown to improve social cognition in older adults [54] and reduce negative behavioral symptoms in patients with frontotemporal dementia [55]. Additionally, OXT treatment can reverse anxiety and social dysfunction in offspring exposed to prenatal stress [56, 57]. Importantly, OXT influences social behavior by modulating dopaminergic neurons, particularly in the VTA [58–60]. This interaction enhances the salience of social stimuli, whether it is positive or negative. These findings underscore the importance of OXT-DA interactions in both normal and pathological social behaviors, providing insights into potential therapeutic targets for social deficits. These lines of evidence suggest that OXT plays a vital role in mitigating the adverse effects of stress; however, despite its effectiveness in reducing stress-induced anxiety and improving social behaviors, the specific mechanisms by which OXT counteracts dopamine dysfunction under stress are not yet fully understood.

In the present study, we demonstrated that OXT pretreatment significantly reduces the effects of CORT by increasing PKAc-α levels and enhancing CREB phosphorylation at Ser 133. PKA is a holoenzyme that remains inactive under basal conditions. However, when activated, its active form, PKAc-α, transfers phosphate groups to Ser/Thr residues of target proteins, including CREB [61]. In PC12 cells, CREB phosphorylation facilitates TH gene expression through CREB/AP-1 family members [50], and OXT treatment increases TH protein levels in the adrenal medulla of socially isolated rats without affecting TH mRNA levels [62]. Previous research has shown that OXT can prevent cell death from metabolic stress by enhancing the MAPK/ERK-CREB signaling pathway in pancreatic beta cells [63]. Additionally, OXT promotes long-term synaptic plasticity and memory via MAPK cascade activation and CREB phosphorylation in the hippocampus, which are crucial for offspring development and survival [64, 65]. Taken together, these findings suggest that OXT may modulate dopaminergic function in SH-SY5Y cells by influencing PKAc-α, pCREB, and pTH levels to counteract the adverse effects of CORT. Furthermore, the protective effects of OXT were abolished by pretreatment with H89, a potent and selective PKA inhibitor [66], indicating that the protective effects of OXT operate through the PKA/CREB pathway.

Finally, the finding that atosiban reversed the protective effects of OXT against CORT toxicity suggested that OXT exerts its protective effect through OXT receptors, emphasizing its role in mitigating CORT toxicity. Indeed, the expression of OXT receptors in SH-SY5Y cells has been documented in various studies. For instance, one study demonstrated that activation of OXT receptors in these cells can modulate the expression of synaptic adhesion molecules, confirming the presence of functional receptors [67]. Additionally, oxytocin affects neurite outgrowth and cytoskeletal protein expression, further supporting the presence of OXT receptors in SH-SY5Y cells [68]. These findings collectively support the notion that SH-SY5Y cells express oxytocin receptors and respond to oxytocin signaling.

**Fig. 7:**
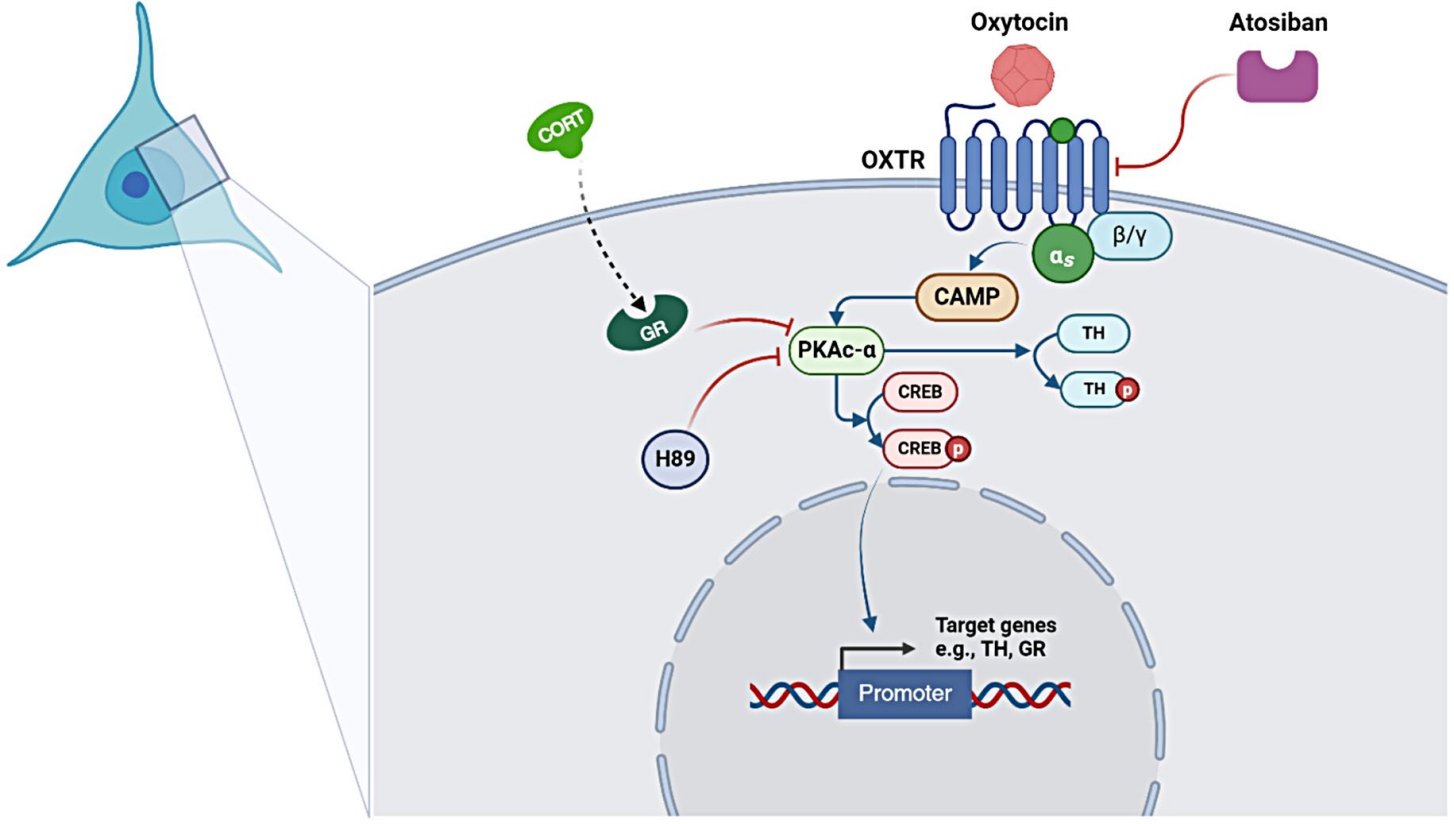
Proposed mechanism underlying the neuroprotective effects of OXT against CORT-induced dopamine dysfunction in SH-SY5Y cells. OXT binds to its receptor, resulting in augmented stimulation of cAMP, which serves as an activator of PKA. Subsequently, PKA phosphorylates TH at Ser40 and CREB at Ser133. Phosphorylated CREB can bind to downstream coactivators to orchestrate the transcriptional activity of its target, including the TH gene. This regulatory process contributes to the protective effects of OXT against CORT-induced toxicity, thereby conferring neuroprotection. (Created with BioRender.com/Mahidol University)

Our findings suggest a potential role for OXT in counteracting stress-induced DA dysfunction through the PKA/CREB signaling pathway (see Fig. 7). By showing that OXT increases PKAc-α levels and enhances CREB phosphorylation, with these effects blocked by atosiban and the PKA inhibitor H89, our study highlights OXT’s potential to influence dopaminergic signaling. While our results imply that OXT could mitigate stress-related DA dysregulation, we recognize that these findings provide indirect rather than direct evidence of its role in dopaminergic regulation. Future research should include functional assays to clarify this mechanistic link. Nonetheless, the ability of OXT to modulate DAergic activity and potentially enhance stress resilience suggests promising therapeutic strategies for stress-related disorders, where DA dysfunction is a key factor. This insight advances our understanding of stress neurobiology and may inform novel approaches for improving mental health and cognitive function in stress-prone conditions.

### Limitations

There are some limitations to this study that should be considered. First, in this study, the undifferentiated SH-SY5Y cells were used as a model system to study dopaminergic function. Although these cells express certain dopaminergic markers, they do not fully replicate the morphology and functionality of mature dopaminergic neurons due to their tumor cell origin. Utilizing differentiation protocols could improve the relevance of SH-SY5Y cells as a model for studying dopaminergic mechanisms. Consequently, future studies should incorporate specific differentiation protocols or use complementary models, such as primary neurons or animal models, to achieve a more comprehensive understanding of oxytocin’s effects on dopaminergic regulation.

Another limitation of this study is its reliance on Western blotting as the primary method for assessing protein expression and phosphorylation levels. While Western blotting provides valuable information about protein dynamics, it has limitations in sensitivity and quantification. Incorporating additional techniques, such as ELISA or mass spectrometry, could yield more comprehensive and precise data, allowing for better validation of our findings and potentially uncovering subtler variations in protein expression and modifications. Future studies that integrate these complementary methods would strengthen the robustness and accuracy of the data, advancing our understanding of oxytocin’s role in dopaminergic regulation.

## Conclusion

In conclusion, our study provides preliminary evidence that OXT may plays a role in mitigating the adverse effects of stress on DA function, potentially through the PKA/CREB signaling pathway. Although these findings suggest that OXT could be a promising therapeutic intervention for stress-related disorders, further research using differentiated cellular models and complementary techniques is necessary to confirm and expand upon these results. Our study contributes to the understanding of the neurobiological mechanisms underlying stress resilience and suggests potential pathways for developing targeted treatments that leverage the neuroprotective properties of OXT to address disorders characterized by DA dysregulation and impaired stress responses.

## Data Availability

No datasets were generated or analysed during the current study.
